# Evaluation of a Hands-On Wrist Fracture Simulator for Fracture Management Training in Emergency Medicine Residents

**DOI:** 10.7759/cureus.27030

**Published:** 2022-07-19

**Authors:** Nathan Olson, Joseph Griggs, Kamna S Balhara, Kristen Kann, Michael D April, Adriana S Olson

**Affiliations:** 1 Emergency Medicine, University of Chicago, Chicago, USA; 2 Emergency Medicine, Bayne-Jones Army Community Hospital, Fort Polk South, USA; 3 Emergency Medicine, Johns Hopkins University School of Medicine, Baltimore, USA; 4 Emergency Medicine, Brooke Army Medical Center, Fort Sam Houston, USA; 5 Emergency Medicine, San Antonio Uniformed Services Health Education Consortium (SAUSHEC), Fort Sam Houston, USA

**Keywords:** closed fracture reduction, fracture reduction, orthopedic fractures, emergency medicine resident, simulation trainer, distal radius fracture management

## Abstract

Background

Fractures are common in the emergency department, and fracture management training poses certain challenges. Recent emergency medicine (EM) residency graduates feel only somewhat prepared to manage fractures. In this study, our objectives were to determine the effect of introducing a wrist fracture simulator (Sawbones®) to traditional EM fracture management education and to assess resident attitudes, comfort with fracture management, and perceptions of the simulator.

Methodology

This six-month prospective study involved postgraduate year one residents at two academic EM programs. For convenience, each residency was considered as one test group. One residency group was deemed the traditional group (n = 10), while the other was the intervention simulator group (n = 16). Identical traditional lectures and buddy splinting workshops were provided. The simulator group received supplemental training with the Sawbones® simulator. Groups were filmed using this simulator for fracture management before the teaching sessions and at six months. Grading utilized a 27-point scale, with a subscale covering reduction. Data were collected regarding attitudes, comfort with fracture management, and perceptions of the simulator.

Results

In total, 26 residents participated in the study. There was no significant difference between groups at six months in overall fracture management scores (traditional group: 15.8 ± 3.1; simulator group: 15.4 ± 3.9; p = 0.92). On the subscale of fracture reduction skills, the simulator group showed significant improvement (p = 0.0078), while the traditional training group did not (p = 0.065). Both groups reported satisfaction with the simulator, improved comfort, and knowledge of fracture management.

Conclusions

Fracture management is an essential competency, and prior research has shown that most graduating EM residents do not feel comfortable with these skills. All participating residents in this study struggled with adequate fracture management, even after the teaching session. Our study suggests that there is a benefit to supplementing traditional training with a fracture simulator.

## Introduction

Fractures are frequently managed in the emergency department (ED), and it is estimated that fracture rates will increase with a more active and aging population [1]. Specifically, distal radius fractures are commonly managed by emergency physicians [2,3]. Orthopedic education and training for emergency medicine (EM) residents are lacking [4]. Recent EM residency graduates reported feeling only somewhat prepared to care for fractures in the ED [5]. The Accreditation Council for Graduate Medical Education (ACGME) has no specific milestones addressing competency in fracture management skills [6]. Additionally, in a cognitive assessment of common upper extremity disorders, EM residents struggled to reach competencies [7]. The challenges of attaining competency in orthopedic management extend beyond EM residency programs and have similarly been seen in internal medicine, radiology, and pediatric programs [7-9]. Furthermore, the pervasive issue of inadequate musculoskeletal education has been documented in medical school, compounding these training challenges [10,11]. Further stressing the need for adequate orthopedic training in EM residents is the shortage of orthopedic surgery on-call coverage. Only one-third of emergency physicians reported they worked at hospitals with full-time orthopedic surgery coverage, and 8% stated they never had orthopedic coverage when working in the ED [12]. Orthopedic training interventions have shown promising results among both EM and orthopedic physicians [13,14]. Adequate fracture management training is essential for improving patient outcomes and has been shown to decrease the need for surgical intervention [14,15]. An area requiring further study in EM is orthopedic simulator training to augment traditional education. Fracture simulators have been successfully implemented in orthopedic residency training programs [16]. Simulation education, when compared to traditional medical training, has been shown to enhance the quality and outcomes of advanced cardiac life support, improved surgical skills, medical team communication, and central venous access [17-20]. In addition, the vast majority (94%) of resident physicians report wanting simulation used for procedure teaching [21]. However, there is a lack of research evaluating the use of fracture simulators for EM resident education. In this study, we sought to assess the effects of integrating a novel wrist fracture simulator into traditional fracture management training. Our primary objective was to determine the effect of introducing a novel wrist fracture simulator (Sawbones®) to traditional EM fracture management education for postgraduate year (PGY)-1 EM residents. The secondary objectives were to assess PGY-1 EM resident attitudes and comfort levels with fracture management and perceptions of the simulator.

## Materials and methods

This study was conducted among PGY-1 EM residents at two ACGME-accredited three-year programs at tertiary care centers. Both sites obtained institutional review board approval. The study subjects were PGY-1 EM residents at both sites. There were no further exclusion criteria for participation in the study. Site 1 had 16 EM PGY1 residents (simulator group) and site 2 had 10 EM PGY1 residents (traditional training group). Informed consent was obtained from all study participants. EM PGY1s at both sites completed a demographic form and pre-assessment survey asking about prior orthopedic experience and comfort and attitudes toward fracture management. All participants at both institutions were filmed performing fracture management on a dorsally displaced distal radius fracture using a Sawbones® fracture simulator arm [22]. After initial filming, both groups received identical traditional training on fracture management that included a lecture and a hands-on buddy splinting workshop. Participants in the simulator group received an additional 30-minute hands-on workshop specifically focused on fracture reduction techniques using the Sawbones® wrist fracture simulator. All participants completed a post-teaching survey regarding their attitudes and perceptions toward the teaching session and their comfort levels with fracture management immediately following their educational sessions. Six months post-teaching session, all residents were again filmed performing fracture management on a distal radius fracture using the Sawbones® simulator. After filming, all participants completed a six-month post-training survey regarding their attitudes and perceptions toward the simulator and their comfort levels with fracture management. A standardized fracture management assessment checklist for grading the video recordings was created in a multi-step approach (Supplemental Appendix) The checklist focused on the core elements of emergency fracture management, including assessment, reduction, and stabilization. First, a literature review was conducted using EM and orthopedic primary source material and was used to create a list of required fracture management actions [23-25]. A panel of five attending emergency physicians at both sites then voted on which items should be included, modified, or removed from the initial checklist. There had to be a group consensus for each item on the checklist for it to remain. Four rounds of voting and modification took place to reach a final checklist consensus. Items 7-11 on the checklist specifically focused on fracture reduction actions rather than fracture assessment or splint application. The two filmed sessions of each EM resident’s fracture management skills were viewed and scored independently by two attending emergency physicians (one from each site) who used the grading assessment checklist to score each video session. Two additional attending emergency physicians watched the filmed assessments and adjudicated any grading discrepancies. Reliability assessments were completed on the fracture management checklist using the multi-grader assessments. The internal reliability of the checklist was assessed by computing Cronbach’s alpha. Inter-rater reliability for the total score and items 7-11 (fracture reduction subscore) were assessed using the Shrout-Fleiss intraclass correlations for continuous variables calculation. The p-values for the demographics, pre-teaching, and six-month post-teaching surveys were calculated using Fisher’s exact test or Wilcoxon test. The p-values for pre and six-month post-training video scores were calculated using the Student’s t-test or Wilcoxon sign-rank test. All p-values were set at <0.05 for statistical significance.

## Results

A total of 26 EM PGY1s participated in this study. There were 16 residents in the simulator group and 10 residents in the traditional training group. There were no statistically significant differences in pre-assessment demographic information between the groups (Table 1).

**Table 1:** Demographics and pre-training survey comparisons. ^1^Likert scale 1-5. IQR: interquartile range

	Simulator (n = 16)	Traditional training (n = 10)	P-value
Age (years), median (IQR)	28.0 (27.0-30.0)	27.0 (26.0-29.0)	0.387
Sex (male), n (%)	12 (75%)	8 (80%)	0.999
Spent prior time as a physician, n (%)	3 (19%)	0 (0%)	0.262
Completed orthopedics rotation, n (%)	5 (31%)	3 (30%)	0.999
Previously involved/observed fracture reduction, n (%)	8 (50%)	7 (70%)	0.428
Number of reductions involved/observed, median (IQR)	0.5 (0.0-9.0)	3.0 (0.0-7.0)	0.684
Prior splint/cast experience, n (%)	11 (69%)	7 (70%)	0.999
Number of splint/casts involved, median (IQR)	2.5 (0.0-5.5)	2.0 (0.0-2.0)	0.411
Procedure simulator experience, n (%)	7 (44%)	4 (40%)	0.999
Comfort level with orthopedic reductions,^1^ median (IQR)	1.5 (1.0-2.0)	1.5 (1.0-2.0)	0.931
Comfort level with splinting,^1^ median (IQR)	2.0 (1.0-2.5)	2.0 (1.0-3.0)	0.999

Post-training surveys immediately following the teaching session revealed no significant differences in satisfaction with teaching sessions or improved knowledge and comfort with reduction and splinting between the two groups (Table 2). However, both groups were satisfied with the Sawbones® simulator and rated it similar to a human fracture and felt that the teaching sessions improved their knowledge and comfort levels with fracture management skills (Table 2). Six-month post-training survey results are shown in Table 2. In the six-month post-training survey, there was a statistically significant difference in the number of orthopedic rotations completed by the interns in the traditional training group (40%) versus the simulator group (0%) (p = 0.014) (Table 2). Residents in both groups had decreased comfort levels with orthopedic reductions and splinting at six months compared to their comfort levels immediately after training.

**Table 2:** Attitudes and perceptions of EM PGY1 residents. ^1^Likert scale 1-5. IQR: interquartile range; EM: emergency medicine; PGY1: postgraduate year one

	Simulator (n = 16)	Traditional training (n = 10)	P-value
Immediate post-training survey^1^			
Comfort level with orthopedic reductions,^1^ median (IQR)	4.0 (2.0-4.0)	3.5 (2.0-4.0)	0.879
Comfort level with splinting,^1^ median (IQR)	4.0 (2.0-4.0)	4.0 (4.0-4.0)	0.252
Satisfaction with teaching session,^1^ median (IQR)	5.0 (4.0-5.0)	5.0 (5.0-5.0)	0.197
Improved knowledge of reduction,^1^ median (IQR)	5.0 (4.0-5.0)	5.0 (4.0-5.0)	0.582
Improved knowledge of splinting,^1^ median (IQR)	5.0 (5.0-5.0)	5.0 (4.0-5.0)	0.269
Improved comfort with reduction,^1^ median (IQR)	4.0 (4.0-5.0)	4.0 (4.0-5.0)	0.936
Improved comfort with splinting,^1^ median (IQR)	4.0 (4.0-5.0)	5.0 (4.0-5.0)	0.136
Six-month post-training survey^1^			
Comfort level with orthopedic reductions,^1^ median (IQR)	3.5 (2.0-4.0)	3.0 (2.0-4.0)	0.934
Comfort level with splinting,^1^ median (IQR)	3.5 (2.5-4.0)	4.0 (4.0-4.0)	0.183
Satisfaction with simulator,^1^ median (IQR)	4.0 (3.5-5.0)	4.0 (3.0-5.0)	0.638
Sawbones® similarity to human fracture,^1^ median (IQR)	4.0 (3.0-4.0)	4.0 (3.0-4.0)	0.286
Involvement in casting since training, n (%)	6 (38%)	6 (60%)	0.422
Involvement in splinting since training, n (%)	7 (44%)	6 (60%)	0.688
Number of reductions/splinting casting involved/observed since training, n (%)	0.0 (0.0-1.5)	2.0 (0.0-8.0)	0.059
Completed orthopedic rotation since training, n (%)	0 (0%)	4 (40%)	0.014

There was no statistically significant difference in overall fracture management scores or in the subscale of fracture reduction skills between groups at six months post-training (p = 0.807 and p = 0.461, respectively) (Table 3). There was, however, a statistically significant increase from pre-training to six-month post-training total scores for both the simulator and traditional training groups (p < 0.0001 and p = 0.0004, respectively) (Table 4). There was a statistically significant increase from the pre-training scores to six-month post-training scores for fracture reduction-specific items 7-11 for the simulator group (p = 0.0078), but not for the traditional training group (p = 0.063) (Table 4). The checklists demonstrated internal reliability using Cronbach’s alpha for the post-training videos total score of 0.63 and for items 7-11 of 0.67. Inter-rater reliability for the video checklist was excellent, with a Shrout-Fleiss intraclass correlation of 0.78 and 0.75 for the total score and items 7-11 score.

**Table 3:** Fracture management skills. ^1^Subscale of fracture reduction skills. SD: standard deviation

	Simulator (n = 16)	Traditional training (n = 10)	P-value
Six-month post-training scores			
Total score, mean ± SD	15.4 ± 3.9	15.8 ± 3.1	0.807
Items 7-11,^1^ mean ± SD	2.6 ± 1.3	2.1 ± 2.1	0.461

**Table 4:** Comparison of pre-training scores and six-month post-training scores at each site. ^1^Subscale of fracture reduction skills. SD: standard deviation

	Pre-training scores	Six-month post-training scores	P-value
Simulator group (n = 16)			
Total score, mean ± SD	8.9 ± 4.0	15.4 ± 3.9	<0.0001
Items 7-11,^1^ mean ± SD	1.4 ± 0.9	2.6 ± 1.3	0.008
Traditional training group (n = 10)			
Total score, mean ± SD	9.5 ± 3.7	15.8 ± 3.1	0.0004
Items 7-11,^1^ mean ± SD	0.8 ± 1.3	2.1 ± 2.1	0.063

## Discussion

Fractures, especially distal radius fractures are commonly treated by EM physicians who have expressed discomfort with their management [1-3,5].

As expected in our study, there was a statistically significant increase in procedural competency from the initial pre-training to post-training video assessments for all participants. While there was no significant difference in the overall scores between the simulator and traditional training group residents, the subgroup analysis for items 7-11 (fracture reduction skills) showed a statistically significant increase in assessment scores for the simulator group compared to no significant increase in assessment scores for the traditional training group. These items (7-11) correlate specifically with the technical aspects of fracture reduction that were the focus of the additional 30 minutes of hands-on practice with the Sawbones® simulator that the simulator group received. Gaining adequate levels of hands-on practice with real or realistic fractures has traditionally been an area where EM orthopedic education has struggled. It is crucial as adequate fracture reduction has been shown to decrease surgical requirements for distal wrist fractures [13-15]. This was also seen in this study with the low number of reported reduction/splinting/casting experiences both study groups were involved with (Table 2).

While there was no difference in the overall scores between the groups, the finding that the simulator group showed significant improvement in reduction-specific maneuvers while the traditional training group did not is important. Fracture injury assessment and splinting skills were expected to be similar between the groups as they shared common traditional training for these skills. This finding is especially notable considering that 40% of the traditional training group completed an orthopedic rotation during the course of our study compared to no residents in the simulator group. The traditional training group, likely because of their early exposure to an orthopedic rotation, reported significantly more reduction/splinting opportunities during the six-month period between study training and assessment. Our study demonstrates a potential benefit with respect to fracture reduction skills of the addition of a fracture reduction simulator to traditional EM orthopedic training.

One interesting result from the study was lower than expected overall scores on the post-training assessments for both groups. The traditional and simulator groups scored 15.8 ± 3.1 and 15.4 ± 3.9, respectively, on the 27-point assessment. While both groups did show a significant improvement from their pre-training scores, their post-training scores likely do not reflect reaching a level of adequate competency for independent fracture management practice. This is likely multifactorial including knowledge and skill degradation during the six-month gap between training and post-training assessment. In addition, this was compounded by the limited number of fracture management opportunities the residents had during the six-month study period. This also likely corresponds to the decrease in six-month comfort levels, with reductions seen in both groups and lack of comfort also found in recent EM graduates [5]. As such, these findings highlight the challenges in EM orthopedic training that procedure simulators may help address.

Finally, the EM residents involved in the study were satisfied with the simulator and deemed that the Sawbones® simulator was similar to a human fracture. This is similar to prior research from orthopedic surgery literature suggesting that hands-on fracture reduction simulators are deemed realistic and can be reliably used in fracture reduction and casting assessments [16,26-28].

Our study had several limitations. First, some degree of incorporation bias is present. During their training, the simulator group received an additional 30 minutes of time with the Sawbones® model. This likely allowed increased familiarity with the model which could have inflated the fracture reduction benefits noted in the simulator group. However, there were six months between the initial training didactics and the post-assessment which likely helped mitigate this bias. Another limitation was the lack of a validated fracture management checklist for EM residents. Although the checklist created for this study demonstrated strong internal reliability and inter-rater reliability, it would benefit from further validation. Additionally, there was a discrepancy between the completion of an orthopedic rotation between the two groups; 40% of the traditional training group residents completed an orthopedics rotation compared to none in the simulator group during the study period and therefore had more fracture management exposure. This may have caused a negative skew making it harder for the simulator group to show improved reduction skills. There could have possibly been a statistically significant difference between the two groups in overall competency that was masked by this bias.

## Conclusions

Fracture management is an essential skill that most graduating EM residents do not feel comfortable with. Although there was no overall benefit to the introduction of a Sawbones® wrist fracture simulator to traditional EM orthopedic training in procedural competence with fracture management, assessment, reduction, and splinting, there was a benefit in introducing the Sawbones® wrist fracture simulator with respect to fracture reduction skills. Our study, although small, suggests that there is a possible benefit to adding a fracture simulator to supplement traditional EM orthopedic training.

## Appendices

**Table 5:** Fracture management grading checklist.

Number	Item	Incorrect or not done	Done correctly	Additional grader notes
1	Uses all provided appropriate equipment			Stockinette, webril, plaster, ace wrap, water bucket, doesn’t matter order
2	Assesses neurovascular status before reduction			Check pulses- radial/ulnar, sensation- radial/ulnar/median, motor- thumb/finger movement (can verbalize “checking neurovascular exam” for credit)
3	Performs skin exam prior to reduction			Can verbalize “checking skin exam” for credit
4	Measures plaster from within 3 cm of the MCPs on both the dorsal and volar aspects of the hand			Just distal to the MCP’s will count as correctly done but should be on both volar and dorsal sides
5	Uses ≥8 and ≤12 sheets of plaster in splint			They will use two strips of plaster to make their actual practice splints. They can verbalize the ideal number
6	Uses room temp to slightly warm but not hot water			Ok to verbalize or if they use both a hot/cold knob get credit
7	Distraction force: Directs initial force distally (i.e. longitudinal traction applied)- maintains this throughout reduction (Can ask the assistant to help with this)			If they choose to use finger traps, the assistant will hold the fingers
8	Places both hands around the patient's wrist with the thumbs at the base of the fracture site on the dorsal side			
9	Disengagement force: Recreates the fracture deformity or direction			Colles fracture disengagement force should be dorsally directed
10	Reapposition force: Use thumbs to apply reverse injury pressure			Colles fracture-> dorsal to volar pressure is applied to distal fracture segment
11	Holds slight traction on distal radius to ensure reduction is maintained while splint applied			Finger traps or assistant helping
12	Applies stockinette			As first layer
13	Extends stockinette further than plaster proximally and distally			Should extend beyond by about 3 inches – or enough to fold it over plaster
14	Cuts thumb hole cut in the stockinette			
15	Applies 2-3 layers of cotton webril between plaster and stockinette, additional 1-2 layers over bony prominences or pressure points			Webril should be relatively smooth and overlap about 50% of prior roll
16	Folds back stockinette over plaster ends			Can be before or after ace wrap
17	Soaks splint with water until saturated			
18	Squeezes out excess water by running fingers down length of plaster			
19	Smoothes out plaster			Either before or after applied to patient
20	Applies a sugar tong splint			Only sugar tong splint gets credit for this fracture
21	Places plaster just proximal to both dorsal and volar MCPs			
22	Molds plaster until splint has hardened			Credit for verbalizing molding splint until hard
23	Ace wrap placed around cotton/plaster			
24	Secures Ace wrap			Can use tape or metal clips or Velcro
25	Repeats neurovascular exam			Can verbalize this for credit
26	Orders post reduction XR			Can verbalize for credit
27	Places patient in a sling			Can verbalize for credit
